# Association Between Domain-Specific Physical Activity and Novel Inflammatory Biomarkers Among US Adults: Insights From NHANES 2007–2018

**DOI:** 10.1155/mi/1989715

**Published:** 2025-06-24

**Authors:** Xin-ying Liu, Kai Yao

**Affiliations:** ^1^Endoscopy Center, Jinshan Hospital, Fudan University, Shanghai 201508, China; ^2^Department of Neurology, Jinshan Hospital, Fudan University, Shanghai 201508, China

**Keywords:** leisure-time-related physical activity, lymphocytes ratio, NHANES, physical activity, systemic immune-inflammation index, systemic inflammation response index

## Abstract

**Objectives:** The novel inflammatory biomarkers, including systemic immune-inflammation index (SII), systemic inflammation response index (SIRI), and neutrophil-to-lymphocyte ratio (NLR), can contribute to predicting the future risk of various diseases. However, the impact of different physical activity (PA) domains on systemic inflammation remains unclear. The study aims to investigate the relationship between domain-specific moderate-to-vigorous-intensity PA (MVPA) and these inflammatory biomarkers among US adults.

**Methods:** Participants from the US National Health and Nutrition Examination Survey (NHANES) (2007–2018) were included in this study. The Global Physical Activity Questionnaire was used to assess self-reported MVPA. MVPA was categorized into three domains, including occupation-related MVPA (O-MVPA), transportation-related MVPA (T-MVPA), and leisure-time-related MVPA (LT-MVPA). SII, SIRI, and NLR were derived from the complete blood count results obtained at the NHANES Mobile Examination Centers (MEC). Weighted multivariable linear regression and propensity score matching (PSM) were used to examine the relationship between domain-specific MVPA and inflammatory biomarkers. Additionally, stratified and mediation analyses were performed to assess potential effect modifications and mediators in this association.

**Results:** The study included a total of 29,072 participants. Following PSM, weighted multivariable linear regression indicated a negative association between LT-MVPA meeting PA guidelines ( ≥ 150 min/week) and circulating inflammatory biomarkers (*β* = −36, 95% confidence interval [CI]: −47 to −25, *p*  < 0.001 for SII; *β* = −0.09, 95% CI: −0.13 to −0.05, *p*  < 0.001 for SIRI; *β* = −0.08, 95% CI: −0.11 to −0.05, *p*  < 0.001 for NLR, respectively), adjusting for all potential covariates in model 2. Participants engaging in sufficient T-MVPA (≥ 150 min/week) also exhibited lower SII and SIRI levels (*β* = −17, 95% CI: −32 to −2.4, *p*=0.023; *β* = −0.07, 95% CI: −0.11 to −0.03, *p*=0.002). Conversely, O-MVPA showed no significant correlation with any inflammatory biomarkers (all *p*  > 0.05). No significant effect modification was observed in the association between LT-MVPA or T-MVPA and inflammatory biomarkers (SII, SIRI, and NLR). Mediation analysis showed that body mass index (BMI) mediated the relationships between these inflammatory biomarkers and both LT-MVPA and T-MVPA.

**Conclusions:** The impact of different PA domains on systemic inflammation varies significantly. Given the well-established link between chronic inflammation and diseases such as cardiovascular disease (CVD), diabetes, and metabolic disorders, specific recommendations for PA categories should be provided, particularly targeting individuals with high systemic inflammatory responses.

## 1. Introduction

Accumulating evidence underscores the crucial role of chronic inflammation in the pathogenesis of several diseases, including cancer, dementia, cardiovascular disease (CVD), and frailty [1]. Inflammation, accompanied by elevated levels of neutrophils, proinflammatory chemokines, and cytokines, can induce endothelial dysfunction, vascular wall impairment, thrombosis, and atherosclerosis [2, 3]. These outcomes resulted in the formation, instability, and eventual rupture of plaques, and the subsequent cardiovascular events [3–5]. Abnormal neuroinflammation may also promote the process of neurodegenerative diseases, such as Alzheimer's disease (AD), Parkinson's disease (PD), and muscular amyotrophic lateral sclerosis (ALS) [6]. Furthermore, chronic inflammation increases the risk of aging, type 2 diabetes mellitus (T2DM), hyperlipidemia, nonalcoholic fatty liver disease (NAFLD), and obesity [1, 7]. Therefore, targeting inflammation can help prevent the onset and progression of multiple diseases.

Recent studies have introduced innovative indicators based on circulating inflammatory cell counts, including the neutrophil-to-lymphocyte ratio (NLR), systemic inflammation response index (SIRI), and systemic immune-inflammation index (SII). The SII is calculated using the formula platelet count (PC) × neutrophil count (NC)/lymphocyte count (LC) [8], while the SIRI and NLR are calculated as PC × monocyte count (MC)/LC and NC/LC, respectively [9, 10]. These novel biomarkers offer a comprehensive assessment of the balance between inflammation and immunity in the host [8], demonstrating excellent predictive power for disease prognosis, including cancer, infectious diseases, and CVD [11–14]. Compared to traditional inflammatory indicators, these biomarkers have proven to reflect the body's inflammatory state more accurately and comprehensively [11, 15]. Liu et al. [16] reported that SII outperformed NLR, platelet-to-lymphocyte ratio (PLR), and C-reactive protein (CRP) in predicting coronary artery disease occurrence. Moreover, SII was found to independently predict the long-term prognosis in patients with carotid artery stenting (CAS) and better than CRP as an inflammatory prognostic marker [15]. More importantly, recent studies suggested that blood-based inflammatory biomarkers can contribute to predicting the future risk of various diseases in the general population [17, 18]. A 2022 meta-analysis by Ye et al. [19], comprising 152,996 patients, reported that elevated SII levels are associated with an increased risk of future CVDs, including ischemic stroke, hemorrhagic stroke, and myocardial infarction. A significant positive correlation has also been observed between high SII and various diseases prevalence, such as hepatic steatosis [20], hyperlipidemia [21], rheumatoid arthritis [22], all-cause mortality [23], and metabolic syndrome [24]. Jin et al. [11] included 85,154 participants and indicated that people with elevated SII and SIRI had a higher risk of stroke and all-cause death, while higher SIRI was associated with increased MI incidence in subjects under 60 years. Notably, another large-scale Cohort study showed that baseline SII and SIRI were closely correlated with cardiovascular death and all-cause death during 20-year follow-up [25]. Therefore, individuals with high systemic inflammation require increased medical screening frequency to benefit from secondary prevention treatments [26].

Engaging in physical activity (PA) is broadly recognized for its ability to improve overall health and prevent conditions like mental disorders, hypertension, and CVD. PA can typically be categorized into three domains: occupation-related PA (OPA) (e.g., lifting or carrying large loads during work), transportation-related PA (TPA) (e.g., walking or cycling for commuting), and leisure-time-related PA (LTPA) (e.g., recreational activities like running and playing football) [27]. Nonetheless, the contrasting effects of different PA domains on overall health, termed “PA health paradox,” have been highlighted in previous research [28, 29]. LTPA can provide numerous health benefits by improving lipid metabolism, lowering the risk of diabetes and CVD, and reducing all-cause mortality [27, 30]. Conversely, higher levels of OPA have been associated with adverse health outcomes such as CVD, stroke, depression, and all-cause mortality [31]. On the other hand, PA can influence immune cell mobilization, redistribution, and function, offering anti-inflammatory effects in obesity and other chronic inflammatory conditions [32, 33]. Regular PA is considered an effective behavioral strategy that benefits the immune system and helps prevent numerous diseases [34]. Interestingly, recent studies have shown that OPA may not affect, or might even worsen, the body's inflammatory response [35, 36]. Overall, the impact of different PA domains on systemic inflammation remains unclear. Therefore, this study aims to explore the relationship between different PA domains and circulating inflammatory biomarkers (SII, SIRI, and NLR) through a secondary analysis of National Health and Nutrition Examination Survey (NHANES) data.

## 2. Materials and Methods

### 2.1. Study Population

In this study, six cycles of NHANES data (2007–2018) were analyzed. The National Center for Health Statistics (NCHS) uses a multistage, stratified sample strategy in the NHANES survey to evaluate the nutritional status and general health of non-institutionalized civilians older than 2 months in the United States. A number of home interviews, physical exams, and laboratory testing were performed at Mobile Examination Centers (MEC). To ensure nationally representative estimates, NHANES utilized a sophisticated, multistage probability sampling strategy, oversampling non-Hispanic blacks (NHBs), Hispanics, Asians (since 2011), individuals with low incomes (since 1999), and elderly persons. Additionally, the survey has been authorized by the NCHS Ethics Review Committee, and further permission by the Institutional Review Board is not necessary for any second analysis of the survey data [37]. The NHANES data can be accessed on the NHANES website (http://www.cdc.gov/nchs/nhanes.htm). The current study aimed to include participants older than 18 years. Individuals with missing data in the inflammatory biomarkers, PA conditions, and covariates were excluded.

### 2.2. The Assessment of Moderate-to-Vigorous-Intensity PA (MVPA)

The Global Physical Activity Questionnaire was used to assess self-reported MVPA [38]. According to previous research on PA calculation [39, 40], MVPA was defined as any activity that significantly increases heart rate or breathing. MVPA was categorized into three domains in the questionnaire, including occupation-related MVPA (O-MVPA), transportation-related MVPA (T-MVPA), and leisure-time-related MVPA (LT-MVPA). Specifically, activities performed at work, such as carrying or lifting loads and household chores, were classified as O-MVPA. T-MVPA included activities related to commuting, such as those involved in school, work, and shopping. LT-MVPA encompassed leisure activities like sports and fitness, excluding those related to occupation and transportation [27, 41]. Based on the 2018 PA guidelines, adults should participate in at least 75 min of vigorous-intensity PA, or 150 min of moderate-intensity PA, or an equivalent combination per week [42]. Additionally, vigorous-intensity PA minutes were counted as double the minutes of moderate-intensity PA [43]. Consequently, participants in the present study were classified into two groups based on their total MVPA minutes per week: (1) 150 min or more, meeting the 2018 PA guidelines, and (2) less than 150 min.

### 2.3. Definition of Inflammatory Biomarkers and Body Mass Index (BMI)

Referring to previous NHANES literature [44, 45], variables such as the SII, SIRI, and NLR were derived from the complete blood count results obtained at the NHANES MEC. The detailed laboratory methodology for the complete blood count test is available on the NHANES website. PC, NC, MC, and LC were utilized to calculate the biomarkers. The formulas used were SII = PC × (NC/LC), SIRI = PC × (MC/LC), and NLR = NC/LC, as described in previous research [9, 10]. Additionally, weight and height were measured by a digital scale and a stadiometer, respectively, according to standard MEC protocols. The BMI was calculated as body weight (kg)/height (m^2^).

### 2.4. Covariates

Age, gender, education level, race/ethnicity, family income, marital status, smoking status, alcohol use, and past medical histories (including diabetes, hypertension, coronary heart disease (CHD), stroke, asthma, arthritis, chronic bronchitis, and cancer) were all considered as covariates. Based on prior studies and clinical significance, these key variables, which could potentially affect PA and inflammatory biomarkers, were included as confounders [10, 21, 43, 46]. The definitions of covariates were consistent with those reported in previous research [47–49]. Specifically, race/ethnicity was categorized into four groups: non-Hispanic white (NHW), NHB, Hispanic, and other races. Family income was divided into low (less than $20,000 annually) and high (more than $20,000 annually) groups. Marital status was grouped into married or living with a partner and living alone. Three categories were used to classify education levels: fewer than 9 years, 9–12 years, and more than 12 years. Smoking status was defined as never, former, and current smokers [47]. Additionally, past medical histories, including hypertension, diabetes, stroke, CHD, asthma, arthritis, chronic bronchitis, and cancer, were determined based on self-reported questionnaires indicating whether a doctor had previously diagnosed these conditions.

### 2.5. Statistical Analysis

The recommended Taylor procedures and Weights from the NCHS (www.cdc.gov/nchs/nhanes/tutorials) were utilized to manage the intricate survey sample design and generate estimates representative of the US population. Referring to previous NHANES studies [50, 51], the sample weight (MEC2YR) was selected to mitigate bias due to unequal selection probabilities, nonresponse, noncoverage, and location sampling variability. The sample weight, along with the cluster and strata variables derived from demographic data, was applied in our weighted analysis. For normally distributed variables, we used the mean ± standard errors (SEs) for description and the Student's *t*-test to compare factors between the low and high inflammatory biomarker groups. The Chi-square test was employed to compare the percentages of categorical variables. Two weighted multivariable linear regression models were used to determine the relationship between domain-specific MVPA and inflammatory biomarkers. Model 1 was adjusted for age, gender, education level, marital status, family income, and race/ethnicity. Model 2 included additional adjustments for smoking status, hypertension, diabetes, CHD, stroke, asthma, arthritis, chronic bronchitis, and cancer. Furthermore, to reduce selection bias, the propensity score matching (PSM) method, initially introduced by Rubin and Rosenbaum in 1983, was applied. This method relies on counterfactual principles and can enhance the strength of causal inferences in observational studies. Previous studies have demonstrated the effectiveness of PSM in reducing selection bias in retrospective studies [52, 53]. In the present study, PSM with the 1:1 nearest neighbor matching algorithm was utilized to balance the differences between groups with sufficient and insufficient MVPA. All previously mentioned confounding factors were used for matching, and the matching effect was illustrated using a LOVE plot. Subsequently, post-PSM data were reanalyzed to further validate the reliability of the conclusions. Stratified analyses were then performed across age, gender, race/ethnicity, educational level, smoking status, hypertension, CHD, and stroke to identify potential effect modifications on the association between MVPA and inflammatory biomarkers. Interaction effects were evaluated by adding effect modification terms to the models and applying the likelihood-ratio test.

Furthermore, to examine the mediating role of BMI in the association between MVPA and inflammatory biomarkers, mediation analysis was performed using the mediation package in R. Two models were constructed: the mediator model, a logistic regression assessing the relationship between MVPA (exposure) and BMI (mediator); and the outcome model, a logistic regression including both MVPA and BMI to evaluate their effects on inflammatory markers (outcome). The total effect of outcomes was divided into direct and indirect effects, with the mediator factors mediating the indirect effects. According to prior population-based studies with mediation analysis, odds ratios (ORs) and 95% confidence intervals (CIs) for both direct and indirect effects were calculated using the bootstrap method (using nonparametric bootstrapping with 1000 iterations). The mediation percentage, defined as the ratio of the indirect effect to the total effect, represents the mediating effect [54–57].

To ensure the robustness of our results, several sensitivity analyses were performed. First, considering the potential influence of diet, we additionally included the Dietary Inflammatory Index (DII), a measure of dietary inflammatory potential, as a covariate. Second, different MVPA duration thresholds were examined—any MVPA (>0 min/week) and optimal MVPA (≥300 min/week)—to confirm the stability of our conclusions. Third, we applied an alternative PSM matching approach, the “subclass” method, to investigate the association between MVPA and inflammation. All analyses were conducted using R 4.2.1 (http://www.R-project.org, The R Foundation). A *p*-value of <0.05 was considered statistically significant.

## 3. Results

### 3.1. Study Population

**Figure 1:**
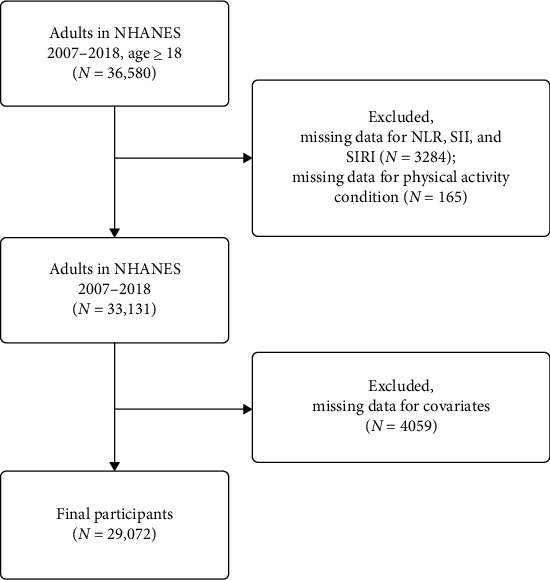
The study's flow diagram. Data from NHANES 2007–2018 for US adults.

The study included 36,580 participants aged over 18 years who completed their interviews between 2007 and 2018. Participants with missing data for inflammatory biomarkers (*n* = 3284), PA (*n* = 165), and covariates (*n* = 4059) were excluded. Consequently, 29,072 individuals were included in this cross-sectional study.  Figure 1 details the inclusion and exclusion process.

### 3.2. Baseline Characteristics

All participants were divided into two quantiles based on their inflammatory biomarker levels (Table 1). This study included 29,072 individuals (mean age, 47.41 years; 47.89% men) from the NHANES 2007–2018. For all inflammatory biomarkers, participants in the high-level groups tended to be older, NHWs, have insufficient LTPA or TPA, higher BMI, be current smokers, and have a history of hypertension, diabetes, stroke, arthritis, chronic bronchitis, and cancer (all *p* < 0.05). Additionally, individuals with higher SII or SIRI levels were more likely to have 9–12 years of education, live alone, and have a history of asthma (all *p*  < 0.05).

### 3.3. Correlation Between Domain-Specific MVPA and Inflammatory Biomarkers

The weighted multivariable linear regression analysis was employed to investigate the correlation between domain-specific MVPA and inflammatory biomarkers. As depicted in Table 2, LT-MVPA meeting recommended guidelines (≥ 150 min/week) showed negative associations with SII, SIRI, and NLR levels (*β* = −39, 95% CI: −48 to −29, *p*  < 0.001; *β* = −0.09, 95% CI: −0.11 to −0.06, *p*  < 0.001; *β* = −0.10, 95% CI: −0.13 to −0.06, *p*  < 0.001, respectively) after fully adjusting for all potential covariates (age, gender, education level, race/ethnicity, family income, marital status, smoking status, hypertension, diabetes, CHD, stroke, asthma, arthritis, chronic bronchitis, and cancer) in Model 2. Participants with sufficient TPA (≥ 150 min/week) also exhibited lower inflammatory biomarker levels (*β* = −19, 95% CI: −31 to −8.4, *p* < 0.001 for SII; *β* = −0.08, 95% CI: −0.11 to −0.05, *p*  < 0.001 for SIRI; *β* = −0.07, 95% CI: −0.10 to −0.03, *p*  < 0.001 for NLR). These findings were consistent in Model 1. Interestingly, O-MVPA was not associated with any inflammatory biomarkers, even when meeting PA guidelines (all *p*  > 0.05).

### 3.4. PSM Analysis

To mitigate selection bias, we employed the PSM method to evaluate the correlation between domain-specific MVPA and inflammatory biomarkers based on MVPA status. LOVE plots showed the matching effect (Figure S1). The demographic characteristics of participants before and after matching were also presented in Tables S1–S3. After matching with full adjustment for all potential covariates, individuals engaging in recommended LT-MVPA (≥ 150 min/week) showed lower inflammatory biomarkers in Model 2 (*β* = −36, 95% CI: −47 to −25, *p*  < 0.001 for SII; *β* = −0.09, 95% CI: −0.13 to −0.05, *p*  < 0.001 for SIRI; *β* = −0.08, 95% CI: −0.11 to −0.05, *p*  < 0.001 for NLR) (Table 3). T-MVPA meeting recommended guidelines (≥ 150 min/week) was also negatively associated with SII and SIRI levels (*β* = −17, 95% CI: −32 to −2.4, *p*=0.023; *β* = −0.09, 95% CI: −0.13 to −0.05, *p*  < 0.001) in Model 2. In contrast, O-MVPA was not correlated with any of the inflammatory biomarkers (all *p*  > 0.05) (Table 3). The results remained robust in Model 1.

### 3.5. Stratified Analyses and Mediation Analysis

**Figure 2:**
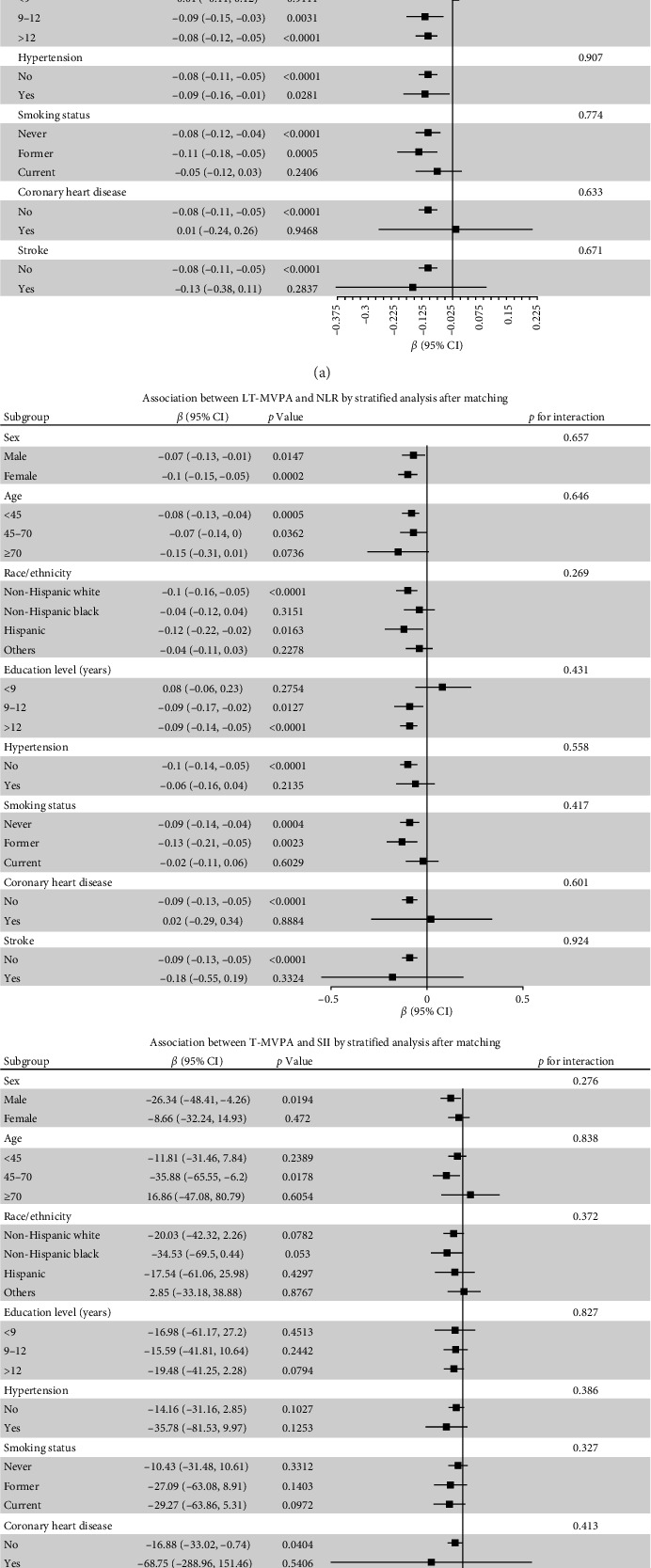
Association between MVPA and inflammatory index by stratified analysis after matching. LT-MVPA, leisure-time moderate-to-vigorous-intensity physical activity; NLR, neutrophil-to-lymphocyte ratio; SII, systemic immune inflammation index; SIRI, systemic inflammation response index; T-MVPA, transportation-related MVPA. Propensity score matching (PSM) was conducted using the 1:1 “nearest” method to balance covariates between groups. Each stratification factor was fully adjusted for all covariates (age, sex, race/ethnicity, education level, marital status, family income, smoking status, hypertension, diabetes, coronary heart disease, stroke, asthma, arthritis, chronic bronchitis, and cancer).

**Figure 3:**
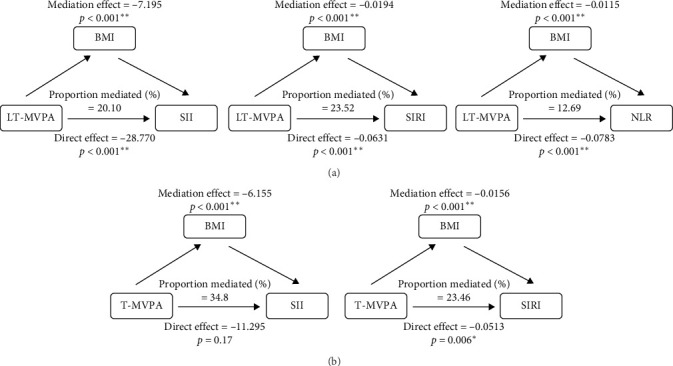
Path diagram of the mediation analysis of BMI on the relationship between MVPA and inflammatory index among US adults in NHANE 2007–2018. (A) Mediation efffect of BMI on the relationship between LT-MVPA and inflammatory index. (B) Mediation efffect of BMI on the relationship between T-MVPA and inflammatory index. *⁣*^*∗*^*p*  < 0.05, *⁣*^*∗∗*^*p*  < 0.001.

Stratified analyses were conducted after PSM to assess potential effect modifications on the association between domain-specific MVPA and inflammatory biomarkers. As shown in Figure 2, no significant effect modification was observed in the association between LT-MVPA (≥ 150 min/week) or T-MVPA (≥ 150 min/week) and inflammatory biomarkers (SII, SIRI, and NLR) (all *p* for interaction < 0.05). Additionally, the mediating effects of BMI on the correlation between LT-MVPA and inflammatory biomarkers were investigated. First, as illustrated in Table 4, recommended LT-MVPA and T-MVPA (≥ 150 min/week) were all negatively associated with BMI (*p*  < 0.001). Conversely, no significant correlation was observed between OPA (≥ 150 min/week) and BMI (*p*  > 0.05). Second, mediation analysis revealed that the mediation effects of BMI on the association between LT-MVPA and inflammatory biomarkers were statistically significant (Figure 3). The mediating ratios were 20.10% for SII, 23.52% for SIRI, and 12.69% for NLR. Furthermore, BMI also mediated the relationships between T-MVPA and inflammatory biomarkers, with mediating ratios of 34.80% for SII and 23.46% for SIRI.

### 3.6. Sensitivity Analyses

First, to account for potential dietary influences, the DII was included in the multivariable linear regression analysis, and PSM analysis was used. LOVE plots demonstrated the matching effect (Figure S2). As shown in Table S4, meeting PA guidelines through LTPA was negatively associated with inflammatory biomarkers in Model 2 after PSM (*β* = −36, 95% CI: −47 to −24, *p*  < 0.001 for SII; *β* = −0.09, 95% CI: −0.13 to −0.05, *p*  < 0.001 for SIRI; *β* = −0.08, 95% CI: −0.11 to −0.04, *p*  < 0.001 for NLR). Similarly, participants achieving ≥150 min/week of T-MVPA had lower inflammatory biomarker levels (*β* = −24, 95% CI: −41 to −6.9, *p*=0.007 for SII; *β* = −0.08, 95% CI: −0.14 to −0.02, *p*=0.009 for SIRI; *β* = −0.09, 95% CI: −0.14 to −0.04, *p*=0.009 for NLR) in Model 2. In contrast, O-MVPA showed no association with inflammatory biomarkers. Second, different PA duration thresholds were examined. Engaging in any amount of LTPA or TPA (> 0 min/week) was associated with lower levels of SII, SIRI, and NLR (*p*  < 0.05). However, O-MVPA, whether at any level (> 0 min/week) or meeting the ≥ 300 min/week threshold, showed no correlation with inflammatory markers (*p*  > 0.05) (Table S5). Third, a different matching approach, the “subclass” method, was conducted to further validate the robustness of the findings. LOVE plots illustrated the matching effect (Figure S3). As illustrated in Table S6, the results remained robust after PSM.

## 4. Discussion

Our study revealed that LT-MVPA following PA guidelines was negatively associated with reduced levels of inflammatory biomarkers (SII, SIRI, and NLR) in US adults. Similarly, individuals who engaged in sufficient T-MVPA (≥ 150 min per week) showed lower levels of SII and SIRI. In contrast, no significant association was found between O-MVPA and these inflammatory biomarkers. Furthermore, mediation analysis indicated that BMI might mediate the relationship between inflammatory biomarkers and both LT-MVPA and T-MVPA. These results suggested that different types of PA influenced systemic inflammation in distinct ways. To the best of our knowledge, this study represented the first demonstration of the PA paradox concerning SII, SIRI, and NLR markers in the US adult population, highlighting how PA types diverge in their inflammatory effects.

Previous studies have reported the relationship between PA and inflammation. Allaouat et al. [58] found that engaging in active commuting for at least 45 min a day was correlated with lower levels of hs-CRP. A cross-sectional study involving 13,748 participants from the NHANES III (1988–1994) demonstrated a significant decrease in serum CRP concentration among individuals participating in light, moderate, and vigorous PA [59]. Another study, which included 796 men and women aged 35–74 years, also indicated that serum proinflammatory interleukin 6 (IL-6) cytokine level was negatively correlated with transportation, leisure time, and total PA. People with higher levels of total PA exhibited lower serum CRP content [60]. Moreover, it was indicated that consistent exercise could lead to reductions in circulating levels of CRP, IL-6, and TNF-α while simultaneously increasing the levels of anti-inflammatory cytokines such as IL-4 and IL-10 [61]. However, the effects of different patterns and intensities of PA on body immunity and inflammation remain elusive. For instance, the association between OPA and inflammation is not well understood. Reuben et al. demonstrated that among elderly individuals aged 70–79, those with higher levels of PA, particularly in house/yard work and recreational activities, exhibited lower plasma CRP concentrations compared to those with low PA levels. The association between between OPA and inflammatory markers IL-6 was not observed [35]. Another cross-sectional study by Feinberg et al. [36] revealed that lower levels of LTPA were associated with a 12% increase in hs-CRP levels, while higher levels of OPA were linked to a 6% rise in hs-CRP. In addition, it was observed that rotating shift workers might have higher risks for systemic inflammation and metabolic issues compared to their day-working counterparts [62]. Puttonen et al. [63] indicated that aggravated systemic inflammation correlated with 2- and 3-shift work, independent of CVD risk factors. Furthermore, the MONICA/KORA study revealed a positive association between job strain and CRP levels [64].In the current study, we found that participants with sufficient LT-MVPA (≥ 150 min/week) had reduced serum inflammatory biomarkers (SII, SIRI, and NLR). T-MVPA meeting the recommended guidelines also showed a negative association with the levels of SII and NLR. Interestingly, there appeared to be no correlation found regarding O-MVPA and circulating biomarkers of inflammation, even when the recommended activity levels for this type were achieved. These findings provide a view that meeting the guidelines for LT-MVPA and T-MVPA could alleviate body inflammation, a benefit seemingly not supplied by O-MVPA.

PA is widely acknowledged as a non-pharmacological, long-term anti-inflammatory intervention, demonstrating strong anti-inflammatory properties along with antioxidant and immune-regulating effects. These benefits influence carbohydrate metabolism, atherosclerosis, and other disease processes [65, 66]. Previous studies indicated that PA could upregulate anti-inflammatory macrophage markers and cytokines (e.g., TGF-β, IL-4, IL-10) and suppress the expression of proinflammatory markers (e.g., IFN-γ, TNF-α, IL-1β) [67, 68]. Additionally, it could help lower overall inflammation by reducing excess adipose mass and improving insulin sensitivity [69]. Another proposed mechanism involves the improvement of endothelial function, given that activated endothelial cells can release cytokines like IL-1, IL-6 and adhesion molecules that influence immune responses [70]. However, emerging evidence indicated that the anti-inflammatory effect of LTPA may not be provided by OPA. The reasons behind this discrepancy—sometimes called the “activity paradox"—remain unclear. One explanation could lie in the differing nature of these activity types. LTPA is generally characterized by activities with conditioning intensity levels, short durations, and sufficient recovery periods, which are beneficial for overall metabolism and cardiorespiratory health. Conversely, OPA often involves unhealthy postures, heavy lifting, and repetitive motions, with little recovery time [71]. Prolonged high-intensity OPA without adequate rest may raise the risks for injuries, knee and hip osteoarthritis, and chronic inflammation, potentially leading to adverse health outcomes [72, 73]. Another contributing factor may be the differential effects on insulin resistance. While LTPA could improve insulin sensitivity, higher levels of OPA showed no such benefits, as indicated by elevated TyG index and HOMA-IR values [74]. Insulin resistance was considered to worsen chronic low-grade inflammation by stimulating the secretion of proinflammatory cytokines [75]. Furthermore, higher-intensity OPA might cause sustained elevations in heart rate during work hours and reduce heart rate variability (HRV) during the following night, indicating impaired autonomic nervous system (ANS) function [76]. This dysfunction could promote various inflammatory diseases by increasing proinflammatory responses and inhibiting anti-inflammatory processes [77].

Importantly, our study highlighted BMI as a pivotal factor in the “PA paradox.” Higher BMI, along with obesity, shows strong connections to chronic low-grade inflammation and different metabolic disorders. Studies in both humans and animals have demonstrated that obesity-induced inflammatory changes in adipose tissue lead to elevated levels of proinflammatory cytokines, including TNF-α, IL-6, and monocyte chemoattractant protein-1 (MCP-1) [78]. Another key contributor to inflammatory response in obesity is organelle dysfunction of metabolic cells. As an example, compared to lean tissues, the liver and adipose tissue in obese individuals exhibit aggravated endoplasmic reticulum (ER) stress and oxidative stress, further exacerbating metabolic deterioration and inflammation [79]. In addition, obesity alters the immune cell composition in adipose tissue by promoting a shift from anti-inflammatory M2 macrophages to proinflammatory M1 macrophages [80]. The gut microbiome also plays a crucial role in obesity-induced inflammation. Changes in the intestinal microbiota in obesity have been linked to increased intestinal permeability, facilitating metabolic endotoxemia and systemic inflammation [81]. These processes further enhanced inflammatory signaling, contributing to the development of metabolic disorders and obesity-related diseases.

To date, the relationship between different PA types and body weight remains unclear, particularly regarding work-related activities. While some studies suggested that high levels of OPA are associated with lower obesity prevalence [82, 83], others reported either no connection or higher obesity risks among active workers [84, 85]. These inconsistencies could stem from the variability of self-reported PA data and variations between job types. It remains unclear whether reverse causation plays a role in this association. To explore this, a longitudinal cohort study tracking over 11,000 participants across 40 years showed no association between OPA and future BMI or weight changes after adjusting for baseline PA levels [86]. In our study, higher levels of LTPA were associated with a lower BMI, whereas no such relationship was observed for OPA. Mediation analysis further indicated that BMI mediated the relationship between PA and inflammatory markers. Occupational activities could influence weight through multiple pathways, including not moving enough during the day, prolonged sedentary behavior, and exposure to poor eating habits. For instance, excessive physical exertion at work may lead to reduced total energy expenditure over 24-h, due to people moving less during non-work hours [87]. Additionally, irregular work schedules and night shifts can disrupt natural sleep patterns, increasing cravings for high-calorie foods and thereby raising energy intake [88]. Many professions requiring extended sitting have been tied to higher body fat and metabolic disorders [89]. Beyond these behavioral aspects, workplace stressors—like high demands, lack of control, and shift rotations—are known to heighten risks for obesity and related conditions. These stressors may disturb the hypothalamic–pituitary–adrenal (HPA) axis and circadian rhythms [90, 91]. Adverse health outcomes caused by high OPA, including mental health challenges like depression or sleep troubles, may also promote weight gain and obesity [92, 93]. In summary, our findings emphasized the crucial role of BMI in explaining the paradoxical relationship between domain-specific PA and health outcomes. More longitudinal studies are needed to further elucidate these mechanisms and confirm our observations.

The strength of our study is the large sample size and the application of novel inflammatory biomarkers. First, the data was derived from a nationwide study involving over 29,000 adult participants, which helps strengthen the reliability of the results. Second, we used novel inflammatory biomarkers (SII, SIRI, and NLR) to overall inflammatory reactions and immune system responses. These biomarkers are effective indicators of the systemic immune and inflammatory state [94]. Compared to traditional inflammatory markers, they provide a more accurate and comprehensive reflection of the body's inflammatory status [11, 15] and are more credible in predicting disease prognosis [95, 96]. Lastly, the association between MVPA and inflammation was evaluated in this study using a PSM method, which yielded estimates that are less biased and more reliable.

Nevertheless, several limitations must be acknowledged. First, a major limitation is the reliance on self-reported PA data, which could result in recall bias and overly optimistic estimates due to social desirability effects. To put it simply, people might not always remember their activities correctly or might report what they think is socially acceptable. For example, more active individuals may overreport their activity, while those with lower PA levels may underestimate it due to recall difficulties. Besides, since PA is commonly considered essential for health, social pressure might lead inactive individuals to exaggerate their activity levels to fit what they believe is healthy. These issues could affect the observed connections between PA and inflammation markers. Therefore, using objective measurement tools, such as accelerometers, pedometers, or wearable devices, would provide more reliable and unbiased PA estimates. Second, the use of NHANES data, which represents the US population, raises concerns about the generalizability of our findings to other populations with different demographic, cultural, and environmental characteristics. Third, there may be unaccounted influences from factors like economic backgrounds, lifestyle habits, and medication history—such as statins or NSAIDs. Another key factor is job category since workplace environment, job characteristics, and occupational stress could alter the impact of PA on body inflammation. That is to say, aspects like irregular work hours and sedentary time should be considered in future analyses. Finally, this cross-sectional study cannot establish a causal relationship between MVPA and circulating inflammatory biomarkers. The potential reverse causality may also influence the mediation of BMI on this association. Longitudinal studies are needed to further investigate the effects of PA on systemic inflammation and the mediated role of BMI, thereby providing stronger evidence for causal inference.

## 5. Conclusion

Our research offers new findings regarding how different PA types impact systematic inflammation in US adults. Importantly, both LT-MVPA and T-MVPA showed clear benefits against inflammation, while similar positive effects were not seen in O-MVPA. These observations suggested that inflammation might help explain the PA health paradox, providing a possible mechanism for the observed discrepancies in health outcomes across different PA domains. From a public health viewpoint, these results emphasized the value of LT-MVPA and T-MVPA as useful approaches for reducing body inflammation and preventing diseases linked to chronic inflammation. Since we know chronic inflammation connects to conditions like CVD, diabetes, and metabolic disorders, adding these activity types to public health guidelines can help lower disease risks, particularly among groups with higher risk of chronic inflammation. To encourage participation in these beneficial activities, policymakers should focus on creating supportive environments—for instance, building safe walking paths, creating cycling lanes, and spaces for recreation. On the other hand, the potential negative impacts of work-related PA on inflammation, weight gain, and overall health deserve more attention. Given the limited inflammation-reducing benefits from work-related activities, promoting diverse activity types—especially leisure time and TPA—among working adults may help balance out these drawbacks. Additionally, workplace initiatives aimed at improving physical conditions and reducing harmful elements, such as long periods of sitting, can contribute to better health outcomes. Future health recommendations should offer specific advice on activity patterns for different populations, particularly those with elevated inflammation levels. More longitudinal studies are needed to better understand the mechanisms behind these connections and to develop targeted strategies for maximizing health benefits from PA.

## Figures and Tables

**Table 1:** Demographic characteristics of participants according to plasma inflammatory biomarker level, NHANES, 2007–2018.

Variables	Total	SII		*p*		SIRI		*p*		NLR		*p*
		Low (≤455.94)	High (>455.94)			Low (≤1.02)	High (>1.02)			Low (≤1.93)	High (>1.93)	
No.	29,072	14,525	14,547	—		14,527	14,545	—		14,527	14,545	—
Occupation-related MVPA: achieved	9911 (37.70%)	4943 (37.74%)	4968 (37.67%)	0.93		4806 (36.38%)	5105 (38.91%)	0.002*⁣*^*∗*^		4962 (37.96%)	4949 (37.47%)	0.54
Transportation-related MVPA: achieved	4018 (12.65%)	2112 (13.31%)	1906 (12.03%)	0.013*⁣*^*∗*^		2150 (13.62%)	1868 (11.76%)	<0.001*⁣*^*∗∗*^		2116 (13.28%)	1902 (12.07%)	0.025*⁣*^*∗*^
Leisure-time MVPA: achieved	9546 (37.78%)	5123 (41.41%)	4423 (34.39%)	<0.001*⁣*^*∗∗*^		5170 (41.82%)	4376 (34.09%)	<0.001*⁣*^*∗∗*^		5076 (41.04%)	4470 (34.76%)	<0.001*⁣*^*∗∗*^
Sex *n* (%)	—	—	—	<0.001*⁣*^*∗∗*^		—	—	<0.001*⁣*^*∗∗*^		—	—	0.08
Male	13,998 (47.89%)	7491 (51.91%)	6507 (44.14%)	—		6291 (43.58%)	7707 (51.82%)	—		6738 (47.11%)	7260 (48.62%)	—
Female	15,074 (52.11%)	7034 (48.09%)	8040 (55.86%)	—		8236 (56.42%)	6838 (48.18%)	—		7798 (52.89%)	7276 (51.38%)	—
Age (years), mean (SE)	47.41 (0.24)	46.74 (0.27)	48.04 (0.27)	<0.001*⁣*^*∗∗*^		45.46 (0.25)	49.19 (0.27)	<0.001*⁣*^*∗∗*^		45.68 (0.25)	49.01 (0.28)	<0.001*⁣*^*∗∗*^
Race/ethnicity, *n* (%)	—	—	—	<0.001*⁣*^*∗∗*^		—	—	<0.001*⁣*^*∗∗*^		—	—	<0.001*⁣*^*∗∗*^
Non-Hispanic white	12,248 (67.43%)	5287 (63.36%)	6961 (71.23%)	—		4837 (60.93%)	7411 (73.36%)	—		5005 (61.56%)	7243 (72.86%)	—
Non-Hispanic black	6030 (10.68%)	3766 (13.77%)	2264 (7.80%)	—		3976 (14.91%)	2054 (6.83%)	—		3928 (14.67%)	2102 (7.00%)	—
Hispanic	2955 (5.61%)	1445 (5.71%)	1510 (5.52%)	—		1480 (5.81%)	1475 (5.42%)	—		1495 (5.99%)	1460 (5.26%)	—
Others	7839 (16.27%)	4027 (17.16%)	3812 (15.45%)	—		4234 (18.35%)	3605 (14.39%)	—		4108 (17.78%)	3731 (14.88%)	—
Education level (year), *n* (%)	—	—	—	<0.001*⁣*^*∗∗*^		—	—	<0.001*⁣*^*∗∗*^		—	—	0.27
<9	2918 (5.14%)	1543 (5.54%)	1375 (4.77%)	—		1431 (5.15%)	1487 (5.13%)	—		1462 (5.27%)	1456 (5.02%)	—
9–12	10,600 (33.01%)	5141 (31.37%)	5459 (34.54%)	—		5074 (30.87%)	5526 (34.96%)	—		5250 (32.41%)	5350 (33.56%)	—
>12	15,554 (61.85%)	7841 (63.09%)	7713 (60.69%)	—		8022 (63.98%)	7532 (59.9%)	—		7824 (62.32%)	7730 (61.41%)	—
Family income, *n* (%)	—	—	—	0.08	—	—	—	0.12		—	—	0.5
Low	7099 (16.96%)	3441 (16.42%)	3658 (17.46%)	—		3391 (16.45%)	3708 (17.42%)	—		3434 (16.76%)	3665 (17.15%)	—
High	21,973 (83.04%)	11,084 (83.58%)	10,889 (82.54%)	—		11,136 (83.55%)	10,837 (82.58%)	—		11,102 (83.24%)	10,871 (82.85%)	—
Marital status, *n* (%)	—	—	—	<0.001*⁣*^*∗∗*^		—	—	<0.001*⁣*^*∗∗*^		—	—	0.57
Married or living with partners	17407 (63.86%)	8901 (65.55%)	8506 (62.28%)	—		8792 (65.01%)	8615 (62.81%)	—		8722 (64.07%)	8685 (63.66%)	—
Living alone	11665 (36.14%)	5624 (34.45%)	6041 (37.72%)	—		5735 (34.99%)	5930 (37.19%)	—		5814 (35.93%)	5851 (36.34%)	—
Body mass index (kg/m^2^), mean (SE)	29.11 (0.08)	28.33 (0.10)	29.84 (0.09)	<0.001*⁣*^*∗∗*^		28.30 (0.10)	29.85 (0.09)	<0.001*⁣*^*∗∗*^		28.54 (0.10)	29.64 (0.10)	<0.001*⁣*^*∗∗*^
Smoking status, *n* (%)	—	—	—	<0.001*⁣*^*∗∗*^		—	—	<0.001*⁣*^*∗∗*^		—	—	<0.001*⁣*^*∗∗*^
Never	16241 (56.03%)	8447 (58.27%)	7794 (53.94%)	—		8873 (60.92%)	7368 (51.57%)	—		8607 (58.59%)	7634 (53.66%)	—
Former	6950 (24.44%)	3380 (24.15%)	3570 (24.7%)	—		3128 (22.73%)	3822 (26.00%)	—		3168 (23.16%)	3782 (25.62%)	—
Current	5881 (19.53%)	2698 (17.58%)	3183 (21.36%)	—		2526 (16.35%)	3355 (22.43%)	—		2761 (18.25%)	3120 (20.72%)	—
Hypertension, *n* (%)	8531 (25.82%)	4013 (23.74%)	4518 (27.76%)	<0.001*⁣*^*∗∗*^		3696 (21.48%)	4835 (29.78%)	<0.001*⁣*^*∗∗*^		3867 (22.55%)	4664 (28.85%)	<0.001*⁣*^*∗∗*^
Diabetes, *n* (%)	3804 (9.77%)	1781 (8.57%)	2023 (10.9%)	<0.001*⁣*^*∗∗*^		1570 (7.25%)	2234 (12.08%)	<0.001*⁣*^*∗∗*^		1615 (7.6%)	2189 (11.78%)	<0.001*⁣*^*∗∗*^
Coronary heart disease, *n* (%)	1171 (3.39%)	547 (3.21%)	624 (3.55%)	0.17		338 (1.89%)	833 (4.74%)	<0.001*⁣*^*∗∗*^		411 (2.42%)	760 (4.28%)	<0.001*⁣*^*∗∗*^
Stroke, *n* (%)	1089 (2.77%)	463 (2.38%)	626 (3.14%)	0.003*⁣*^*∗∗*^		405 (1.95%)	684 (3.53%)	<0.001*⁣*^*∗∗*^		421 (2.11%)	668 (3.39%)	<0.001*⁣*^*∗∗*^
Asthma, *n* (%)	4221 (14.72%)	1938 (13.38%)	2283 (15.96%)	<0.001*⁣*^*∗∗*^		2034 (14.08%)	2187 (15.29%)	0.045*⁣*^*∗*^		2038 (14.18%)	2183 (15.21%)	0.071
Arthritis, *n* (%)	7875 (25.59%)	3671 (23.77%)	4204 (27.29%)	<0.001*⁣*^*∗∗*^		3454 (22.25%)	4421 (28.63%)	<0.001*⁣*^*∗∗*^		3552 (23.02%)	4323 (27.96%)	<0.001*⁣*^*∗∗*^
Chronic bronchitis, *n* (%)	1676 (5.67%)	702 (4.89%)	974 (6.4%)	<0.001*⁣*^*∗∗*^		709 (4.98%)	967 (6.30%)	<0.001*⁣*^*∗∗*^		721 (5.07%)	955 (6.23%)	0.002*⁣*^*∗*^
Cancer, *n* (%)	2748 (10.10%)	1212 (9.32%)	1536 (10.83%)	0.004*⁣*^*∗∗*^		1032 (8.17%)	1716 (11.86%)	<0.001*⁣*^*∗∗*^		1073 (8.23%)	1675 (11.83%)	<0.001*⁣*^*∗∗*^

*Note*: Values are presented as mean (SE) for continuous and frequency (percent) for categorical variables, respectively.

Abbreviations: MVPA, moderate-to-vigorous-intensity physical activity; NLR, neutrophil-to-lymphocyte ratio; SII, systemic immune inflammation index; SIRI, systemic inflammation response index.

*⁣* ^*∗*^ *p*  < 0.05.

*⁣* ^*∗∗*^ *p*  < 0.01.

**Table 2:** Association between domain-specific MVPA and inflammatory biomarkers before matching, NHANES, 2007–2018.

Variables	*β* (95% CI)			
	Model 1	*p*-Value	Model 2	*p*-Value
SII				
Occupation-related MVPA				
Did not achieve	Ref		Ref	
Achieved	−4.9 (−16 to 5.8)	0.37	−6.9 (−18 to 3.8)	0.20
Transportation-related MVPA				
Did not achieve	Ref		Ref	
Achieved	−23 (−34 to −12)	<0.001*⁣*^*∗∗*^	−19 (−31 to −8.4)	<0.001*⁣*^*∗∗*^
Leisure-time MVPA				
Did not achieve	Ref		Ref	
Achieved	−46 (−55 to −37)	<0.001*⁣*^*∗∗*^	−39 (−48 to −29)	<0.001*⁣*^*∗∗*^
SIRI				
Occupation-related MVPA				
Did not achieve	Ref		Ref	
Achieved	−0.01 (−0.04 to 0.01)	0.36	−0.02 (−0.04 to 0.01)	0.16
Transportation-related MVPA				
Did not achieve	Ref		Ref	
Achieved	−0.10 (−0.13 to −0.07)	<0.001*⁣*^*∗∗*^	−0.08 (−0.11 to −0.05)	<0.001*⁣*^*∗∗*^
Leisure-time MVPA				
Did not achieve	Ref		Ref	
Achieved	−0.11 (−0.14 to −0.09)	<0.001*⁣*^*∗∗*^	−0.09 (−0.11 to −0.06)	<0.001*⁣*^*∗∗*^
NLR				
Occupation-related MVPA				
Did not achieve	Ref		Ref	
Achieved	−0.02 (−0.06 to 0.01)	0.18	−0.03 (−0.06 to 0.01)	0.14
Transportation-related MVPA				
Did not achieve	Ref		Ref	
Achieved	−0.08 (−0.12 to −0.05)	<0.001*⁣*^*∗∗*^	−0.07 (−0.10 to −0.03)	<0.001*⁣*^*∗∗*^
Leisure-time MVPA				
Did not achieve	Ref		Ref	
Achieved	−0.11 (−0.15 to −0.08)	<0.001*⁣*^*∗∗*^	−0.10 (−0.13 to −0.06)	<0.001*⁣*^*∗∗*^

*Note*: Model 1 was adjusted for age, sex, race/ethnicity, education level, marital status, and family income. Model 2 was adjusted for age, sex, race/ethnicity, education level, marital status, family income, smoking status, hypertension, diabetes, coronary heart disease, stroke, asthma, arthritis, chronic bronchitis, and cancer.

Abbreviations: MVPA, moderate-to-vigorous-intensity physical activity; NLR, neutrophil-to-lymphocyte ratio; SII, systemic immune inflammation index; SIRI, systemic inflammation response index.

*⁣* ^*∗∗*^ *p* < 0.001.

**Table 3:** Association between domain-specific MVPA and inflammatory index, after matching.

Variables	*β* (95% CI)			
	Model 1	*p*-Value	Model 2	*p*-Value
SII				
Occupation-related MVPA				
Did not achieve	Ref		Ref	
Achieved	−2.7 (−13 to 8.1)	0.62	−3.7 (−14 to 7.1)	0.49
Transportation-related MVPA				
Did not achieve	Ref		Ref	
Achieved	−16 (−31 to −0.59)	0.042*⁣*^*∗*^	−17 (−32 to −2.4)	0.023*⁣*^*∗*^
Leisure-time MVPA				
Did not achieve	Ref		Ref	
Achieved	−36 (−46 to −25)	<0.001*⁣*^*∗∗*^	−36 (−47 to −25)	<0.001*⁣*^*∗∗*^
SIRI				
Occupation-related MVPA				
Did not achieve	Ref		Ref	
Achieved	−0.01 (−0.05 to 0.03)	0.63	−0.01 (−0.05 to 0.02)	0.51
Transportation-related MVPA				
Did not achieve	Ref		Ref	
Achieved	−0.06 (−0.10 to 0.02)	0.004*⁣*^*∗*^	−0.07 (−0.11 to 0.03)	0.002*⁣*^*∗*^
Leisure-time MVPA				
Did not achieve	Ref		Ref	
Achieved	−0.09 (−0.12 to −0.05)	<0.001*⁣*^*∗∗*^	−0.09 (−0.13 to - −0.05)	<0.001*⁣*^*∗∗*^
NLR				
Occupation-related MVPA				
Did not achieve	Ref		Ref	
Achieved	−0.00 (−0.03 to 0.03)	0.95	0.03 (−0.03 to 0.03)	0.83
Transportation-related MVPA				
Did not achieve	Ref		Ref	
Achieved	−0.04 (−0.09 to 0.01)	0.14	−0.04 (−0.09 to 0.01)	0.10
Leisure-time MVPA				
Did not achieve	Ref		Ref	
Achieved	−0.08 (−0.11 to −0.05)	<0.001*⁣*^*∗∗*^	−0.08 (−0.11 to −0.05)	<0.001*⁣*^*∗∗*^

*Note:* Association between MVPA and inflammatory index after matching. Propensity score matching (PSM) was conducted using the 1:1 “nearest” method to balance covariates between groups. Model 1 was adjusted for age, sex, race/ethnicity, education level, marital status, and family income. Model 2 was adjusted for age, sex, race/ethnicity, education level, marital status, family income, smoking status, hypertension, diabetes, coronary heart disease, stroke, asthma, arthritis, chronic bronchitis, and cancer.

Abbreviations: MVPA, moderate-to-vigorous-intensity physical activity; NLR, neutrophil-to-lymphocyte ratio; SII, systemic immune inflammation index; SIRI, systemic inflammation response index.

*⁣* ^*∗*^ *p*  < 0.05

*⁣* ^*∗∗*^ *p*  < 0.001.

**Table 4:** Association between domain-specific MVPA and BMI, after matching.

Variables	*β* (95% CI)			
	Model 1	*p*-Value	Model 2	*p*-Value
Occupation-related MVPA				
Did not achieve	Ref		Ref	
Achieved	−0.20 (−0.08 to 0.47)	0.16	−0.20 (−0.06 to 0.46)	0.13
Transportation-related MVPA				
Did not achieve	Ref		Ref	
Achieved	−1.2 (−1.6 to −0.86)	<0.001*⁣*^*∗∗*^	−1.2 (−1.6 to −0.88)	<0.001*⁣*^*∗∗*^
Leisure-time MVPA				
Did not achieve	Ref		Ref	
Achieved	−1.7 (−2.0 to −1.4)	<0.001*⁣*^*∗∗*^	−1.7 (−2.0 to −1.5)	<0.001*⁣*^*∗∗*^

*Note*: Propensity score matching (PSM) was conducted using the 1:1 “nearest” method to balance covariates between groups. Model 1 was adjusted for age, sex, race/ethnicity, education level, marital status, and family income. Model 2 was adjusted for age, sex, race/ethnicity, education level, marital status, family income, smoking status, hypertension, diabetes, coronary heart disease, stroke, asthma, arthritis, chronic bronchitis, and cancer.

Abbreviation: MVPA: moderate-to-vigorous-intensity physical activity.

*⁣* ^*∗∗*^ *p*  < 0.001.

## Data Availability

All data sets in this study are available at: https://www.cdc.gov/nchs/nhanes/index.htm.

## Supporting Information

Supporting Information Figure S1: Propensity score matching (PSM) effect evaluated by love plots. PSM was conducted using the 1:1 “nearest” method to balance covariates between groups. Table S1: Demographic characteristics of participants matched by occupation-related MVPA status. Table S2: Demographic characteristics of participants matched by transportation-related MVPA status. Table S3: Demographic characteristics of participants matched by leisure-time MVPA status. Figure S2: Propensity score matching (PSM) effect evaluated by love plots. PSM was conducted using the 1:1 “nearest” method to balance all covariates, including the Dietary Inflammatory Index (DII) between groups. Table S4: Association between domain-specific MVPA and inflammatory index, after matching. Adjusted for all covariates, including the DII. Table S5: Association between domain-specific MVPA and inflammatory index. Different PA duration thresholds were examined. Figure S3: Propensity score matching (PSM) effect evaluated by love plots. PSM was conducted using the 1:1 “subclass” method to balance covariates between groups. Table S6. Association between domain-specific MVPA and inflammatory index, after matching. The 1:1 “subclass” PSM method was used.
