# Supplementary material for: Association Between Domain-Specific Physical Activity and Novel Inflammatory Biomarkers Among US Adults: Insights From NHANES 2007–2018
Source: Mediators Inflamm. 2025 Jun 24;2025:1989715. doi: 10.1155/mi/1989715 (PMC12213052; doi:10.1155/mi/1989715)
Supplement: Supporting Information — Figure S1: Propensity score matching (PSM) effect evaluated by love plots. PSM was conducted using the 1:1 “nearest” method to balance covariates between groups. Table S1: Demographic characteristics of participants matched by occupation-related MVPA status. Table S2: Demographic characteristics of participants matched by transportation-related MVPA status. Table S3: Demographic characteristics of participants matched by leisure-time MVPA status. Figure S2: Propensity score matching (PSM) effect evaluated by love plots. PSM was conducted using the 1:1 “nearest” method to balance all covariates, including the Dietary Inflammatory Index (DII) between groups. Table S4: Association between domain-specific MVPA and inflammatory index, after matching. Adjusted for all covariates, including the DII. Table S5: Association between domain-specific MVPA and inflammatory index. Different PA duration thresholds were examined. Figure S3: Propensity score matching (PSM) effect evaluated by love plots. PSM was conducted using the 1:1 “subclass” method to balance covariates between groups. Table S6. Association between domain-specific MVPA and inflammatory index, after matching. The 1:1 “subclass” PSM method was used. [file 1989715.f1.docx]

**
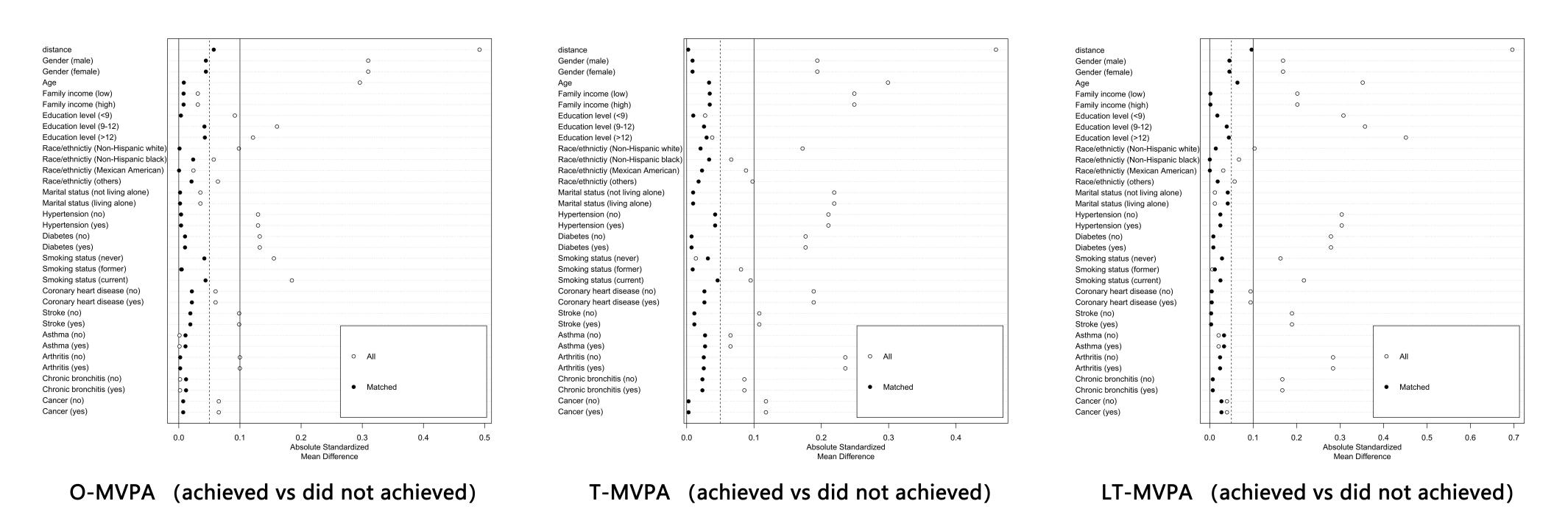
**

**Figure S1.** Propensity score matching (PSM) effect evaluated by love plots. Propensity score matching (PSM) was conducted using the 1:1 "nearest" method to balance covariates between groups. White dots indicate the standard mean diferences before matching, and black dots indicate that after matching. The dashed line represent an standardized mean differences threshold of 1 for evaluating covariate balance. MVPA: moderate-to-vigorous physical activity, O-MVPA: Occupation-related MVPA, T-MVPA: Transportation-related MVPA, LT-MVPA: Leisure-time MVPA.

| **Table S1. Demographic characteristics of participants matched by occupation-related MVPA status** | | | | | | |
| --- | --- | --- | --- | --- | --- | --- |
| **Variables** | **before matching** | | **p** | **after matching** | | **P** |
|  | **Occupation-related MVPA** | |  | **Occupation-related MVPA** | |  |
|  | **Did not achieved** | **Achieved** |  | **Did not achieved** | **Achieved** |  |
| **No.** | 19161 | 9911 |  | 9825 | 9825 |  |
| **Sex, n (%)** |  |  | <0.001** |  |  | 0.02* |
| Male | 8,285 (42.12%) | 5,713 (57.43%) |  | 5,520 (54.77%) | 5,627 (56.96%) |  |
| Female | 10,876 (57.88%) | 4,198 (42.57%) |  | 4,305 (45.23%) | 4,198 (43.04%) |  |
| Age (years), Mean (SE) | 49.19 (0.27) | 44.47 (0.31) | <0.001** | 44.76 (0.29) | 44.63 (0.32) | 0.9 |
| Race/ethnictiy, n (%) |  |  | <0.001** |  |  | 0.37 |
| Non-Hispanic white | 7,594 (65.74%) | 4,654 (70.23%) |  | 4,441 (70.07%) | 4,584 (70.01%) |  |
| Non-Hispanic black | 4,113 (11.32%) | 1,917 (9.63%) |  | 2,052 (10.36%) | 1,906 (9.67%) |  |
| Hispanic | 2,011 (5.81%) | 944 (5.28%) |  | 899 (5.30%) | 941 (5.31%) |  |
| Others | 5,443 (17.13%) | 2,396 (14.86%) |  | 2,433 (14.27%) | 2,394 (15.01%) |  |
| Education level (year), n (%) |  |  | <0.001** |  |  | 0.065 |
| <9 | 2,148 (5.82%) | 770 (4.02%) |  | 723 (3.98%) | 769 (4.05%) |  |
| 9-12 | 6,598 (30.08%) | 4,002 (37.86%) |  | 4,050 (35.23%) | 3,920 (37.26%) |  |
| >12 | 10,415 (64.10%) | 5,139 (58.12%) |  | 5,052 (60.79%) | 5,136 (58.69%) |  |
| Family income, n (%) |  |  | 0.08 |  |  | 0.67 |
| Low | 4,841 (17.39%) | 2,258 (16.24%) |  | 2,287 (15.94%) | 2,235 (16.23%) |  |
| High | 14,320 (82.61%) | 7,653 (83.76%) |  | 7,538 (84.06%) | 7,590 (83.77%) |  |
| Marital status, n (%) |  |  | 0.085 |  |  | 0.92 |
| Married or living with partners | 11,244 (63.22%) | 6,163 (64.91%) |  | 5,950 (64.92%) | 6,113 (65.02%) |  |
| Living alone | 7,917 (36.78%) | 3,748 (35.09%) |  | 3,875 (35.08%) | 3,712 (34.98%) |  |
| Smoking status, n (%) |  |  | <0.001** |  |  | 0.065 |
| Never | 11,169 (58.96%) | 5,072 (51.18%) |  | 5,103 (53.61%) | 5,058 (51.52%) |  |
| Former | 4,613 (24.50%) | 2,337 (24.33%) |  | 2,265 (24.24%) | 2,328 (24.45%) |  |
| Current | 3,379 (16.54%) | 2,502 (24.49%) |  | 2,457 (22.15%) | 2,439 (24.03%) |  |
| Hypertension, n (%) | 6,082 (27.86%) | 2,449 (22.45%) | <0.001** | 2,438 (22.38%) | 2,438 (22.54%) | 0.84 |
| Diabetes, n (%) | 2,861 (11.10%) | 943 (7.59%) | <0.001** | 967 (7.40%) | 943 (7.67%) | 0.53 |
| Coronary heart disease, n (%) | 866 (3.76%) | 305 (2.77%) | <0.001** | 308 (2.45%) | 305 (2.80%) | 0.26 |
| Stroke, n (%) | 828 (3.29%) | 261 (1.93%) |  | 250 (1.69%) | 261 (1.95%) | 0.3 |
| Asthma, n (%) | 2,737 (14.70%) | 1,484 (14.74%) | 0.94 | 1,452 (15.10%) | 1,468 (14.71%) | 0.54 |
| Arthritis, n (%) | 5,537 (27.17%) | 2,338 (22.97%) | <0.001** | 2,378 (22.99%) | 2,327 (23.08%) | 0.91 |
| Chronic bronchitis, n (%) | 1,124 (5.69%) | 552 (5.64%) | 0.89 | 544 (5.34%) | 547 (5.61%) | 0.48 |
| Cancer, n (%) | 1,919 (10.80%) | 829 (8.94%) | <0.001** | 846 (9.23%) | 828 (9.02%) | 0.71 |
| Note: MVPA: moderate-to-vigorous physical activity. P-values were calculated using survey-weighted statistical tests. Propensity score matching (PSM) was conducted using the 1:1 "nearest" method to balance covariates between groups. * P < 0.05, ** P < 0.001   \| **Table S2. Demographic characteristics of participants matched by transportation-related MVPA status** \| \| \| \| \| \| \| \| --- \| --- \| --- \| --- \| --- \| --- \| --- \| \| **Variables** \| **before matching** \| \| **p** \| **after matching** \| \| **P** \| \| **Transportation-related MVPA** \| \| **Transportation-related MVPA** \| \| \| **Did not achieved** \| **Achieved** \| **Did not achieved** \| **Achieved** \| \| **No.** \| 25054 \| 4018 \|  \| 4017 \| 4017 \|  \| \| Sex, n (%) \|  \|  \| <0.001** \|  \|  \| 0.77 \| \| Male \| 11,732 (46.68%) \| 2,266 (56.30%) \|  \| 2,215 (56.72%) \| 2,265 (56.28%) \|  \| \| Female \| 13,322 (53.32%) \| 1,752 (43.70%) \|  \| 1,802 (43.28%) \| 1,752 (43.72%) \|  \| \| Age (years), Mean (SE) \| 48.03 (0.23) \| 43.14 (0.58) \| <0.001** \| 42.61 (0.42) \| 43.15 (0.57) \| 0.3 \| \| Race/ethnictiy, n (%) \|  \|  \| <0.001** \|  \|  \| 0.45 \| \| Non-Hispanic white \| 10,857 (68.50%) \| 1,391 (60.07%) \|  \| 1,499 (61.10%) \| 1,391 (60.09%) \|  \| \| Non-Hispanic black \| 5,146 (10.40%) \| 884 (12.60%) \|  \| 823 (11.50%) \| 884 (12.61%) \|  \| \| Hispanic \| 2,444 (5.31%) \| 511 (7.66%) \|  \| 460 (7.02%) \| 510 (7.62%) \|  \| \| Others \| 6,607 (15.78%) \| 1,232 (19.67%) \|  \| 1,235 (20.38%) \| 1,232 (19.68%) \|  \| \| Education level (year), n (%) \|  \|  \| 0.24 \|  \|  \| 0.53 \| \| <9 \| 2,478 (5.06%) \| 440 (5.70%) \|  \| 407 (5.47%) \| 440 (5.70%) \|  \| \| 9-12 \| 9,053 (32.86%) \| 1,547 (34.08%) \|  \| 1,469 (32.87%) \| 1,547 (34.09%) \|  \| \| >12 \| 13,523 (62.08%) \| 2,031 (60.23%) \|  \| 2,141 (61.66%) \| 2,030 (60.21%) \|  \| \| Family income, n (%) \|  \|  \| <0.001** \|  \|  \| 0.31 \| \| Low \| 5,752 (15.57%) \| 1,347 (26.57%) \|  \| 1,329 (25.03%) \| 1,346 (26.54%) \|  \| \| High \| 19,302 (84.43%) \| 2,671 (73.43%) \|  \| 2,688 (74.97%) \| 2,671 (73.46%) \|  \| \| Marital status, n (%) \|  \|  \| <0.001** \|  \|  \| 0.77 \| \| Married or living with partners \| 15,335 (65.24%) \| 2,072 (54.32%) \|  \| 2,118 (54.81%) \| 2,072 (54.34%) \|  \| \| Living alone \| 9,719 (34.76%) \| 1,946 (45.68%) \|  \| 1,899 (45.19%) \| 1,945 (45.66%) \|  \| \| Smoking status, n (%) \|  \|  \| <0.001** \|  \|  \| 0.32 \| \| Never \| 14,005 (56.11%) \| 2,236 (55.43%) \|  \| 2,269 (57.02%) \| 2,236 (55.45%) \|  \| \| Former \| 6,148 (24.86%) \| 802 (21.54%) \|  \| 836 (21.91%) \| 802 (21.55%) \|  \| \| Current \| 4,901 (19.03%) \| 980 (23.03%) \|  \| 912 (21.07%) \| 979 (23.00%) \|  \| \| Hypertension, n (%) \| 7,668 (26.86%) \| 863 (18.66%) \| <0.001** \| 803 (17.01%) \| 863 (18.66%) \| 0.14 \| \| Diabetes, n (%) \| 3,435 (10.31%) \| 369 (6.08%) \| <0.001** \| 328 (6.26%) \| 369 (6.09%) \| 0.8 \| \| Coronary heart disease, n (%) \| 1,083 (3.67%) \| 88 (1.43%) \| <0.001** \| 61 (1.12%) \| 88 (1.43%) \| 0.22 \| \| Stroke, n (%) \| 1,006 (2.95%) \| 83 (1.59%) \| <0.001** \| 88 (1.45%) \| 83 (1.59%) \| 0.67 \| \| Asthma, n (%) \| 3,693 (14.99%) \| 528 (12.82%) \| 0.011* \| 471 (11.91%) \| 528 (12.82%) \| 0.35 \| \| Arthritis, n (%) \| 7,123 (26.73%) \| 752 (17.73%) \| <0.001** \| 731 (16.76%) \| 752 (17.73%) \| 0.41 \| \| Chronic bronchitis, n (%) \| 1,508 (5.89%) \| 168 (4.18%) \| <0.001** \| 163 (3.71%) \| 168 (4.18%) \| 0.38 \| \| Cancer, n (%) \| 2,489 (10.49%) \| 259 (7.41%) \| <0.001** \| 256 (7.48%) \| 259 (7.41%) \| 0.94 \| \| Note: MVPA: moderate-to-vigorous physical activity. P-values were calculated using survey-weighted statistical tests. Propensity score matching (PSM) was conducted using the 1:1 "nearest" method to balance covariates between groups. * P < 0.05, ** P < 0.001 \| \| \| \| \| \| \| | | | | | | |

| **Table S3. Demographic characteristics of participants matched by leisure-time MVPA status** | | | | | | |
| --- | --- | --- | --- | --- | --- | --- |
| **Variables** | **before matching** | | **p** | **after matching** | | **P** |
|  | **Leisure-time MVPA** | |  | **Leisure-time MVPA** | |  |
|  | **Did not achieved** | **Achieved** |  | **Did not achieved** | **Achieved** |  |
| **No.** | 19526 | 9546 |  | 9452 | 9452 |  |
| **Sex, n (%)** |  |  | <0.001** |  |  | 0.026* |
| Male | 8,783 (44.71%) | 5,215 (53.13%) |  | 4,738 (50.54%) | 5,136 (52.81%) |  |
| Female | 10,743 (55.29%) | 4,331 (46.87%) |  | 4,714 (49.46%) | 4,316 (47.19%) |  |
| **Age (years), Mean (SE)** | 49.59 (0.23) | 43.83 (0.34) | <0.001** | 45.02 (0.27) | 43.98 (0.34) | 0.002* |
| **Race/ethnictiy, n (%)** |  |  | <0.001** |  |  | 0.65 |
| Non-Hispanic white | 8,035 (65.65%) | 4,213 (70.37%) |  | 4,265 (69.56%) | 4,152 (70.20%) |  |
| Non-Hispanic black | 4,144 (11.43%) | 1,886 (9.46%) |  | 1,780 (9.56%) | 1,884 (9.56%) |  |
| Hispanic | 2,090 (5.87%) | 865 (5.18%) |  | 888 (5.21%) | 863 (5.22%) |  |
| Others | 5,257 (17.05%) | 2,582 (15.00%) |  | 2,519 (15.67%) | 2,553 (15.01%) |  |
| **Education level (year), n (%)** |  |  | <0.001** |  |  | 0.036* |
| <9 | 2,446 (6.88%) | 472 (2.28%) |  | 451 (2.57%) | 472 (2.31%) |  |
| 9-12 | 7,979 (38.74%) | 2,621 (23.57%) |  | 2,683 (25.53%) | 2,621 (23.87%) |  |
| >12 | 9,101 (54.38%) | 6,453 (74.15%) |  | 6,318 (71.89%) | 6,359 (73.82%) |  |
| **Family income, n (%)** |  |  | <0.001** |  |  | 0.92 |
| Low | 5,348 (19.50%) | 1,751 (12.77%) |  | 1,739 (12.94%) | 1,746 (12.88%) |  |
| High | 14,178 (80.50%) | 7,795 (87.23%) |  | 7,713 (87.06%) | 7,706 (87.12%) |  |
| **Marital status, n (%)** |  |  | 0.5 |  |  | 0.036* |
| Married or living with partners | 11,737 (64.07%) | 5,670 (63.50%) |  | 5,923 (65.60%) | 5,624 (63.60%) |  |
| Living alone | 7,789 (35.93%) | 3,876 (36.50%) |  | 3,529 (34.40%) | 3,828 (36.40%) |  |
| **Smoking status, n (%)** |  |  | <0.001** |  |  | 0.3 |
| Never | 10,455 (53.03%) | 5,786 (60.97%) |  | 5,723 (59.44%) | 5,713 (60.83%) |  |
| Former | 4,735 (24.54%) | 2,215 (24.28%) |  | 2,210 (24.75%) | 2,194 (24.24%) |  |
| Current | 4,336 (22.44%) | 1,545 (14.75%) |  | 1,519 (15.81%) | 1,545 (14.93%) |  |
| **Hypertension, n (%)** | 6,498 (30.28%) | 2,033 (18.48%) | <0.001** | 1,982 (19.67%) | 2,033 (18.71%) | 0.23 |
| **Diabetes, n (%)** | 3,067 (12.23%) | 737 (5.74%) | <0.001** | 681 (5.61%) | 737 (5.81%) | 0.64 |
| **Coronary heart disease, n (%)** | 919 (3.94%) | 252 (2.48%) | <0.001** | 295 (2.56%) | 251 (2.49%) | 0.81 |
| **Stroke, n (%)** | 917 (3.61%) | 172 (1.39%) | <0.001** | 184 (1.45%) | 172 (1.41%) | 0.85 |
| **Asthma, n (%)** | 2,869 (14.99%) | 1,352 (14.27%) | 0.22 | 1,180 (13.16%) | 1,340 (14.31%) | 0.048* |
| **Arthritis, n (%)** | 6,068 (29.77%) | 1,807 (18.70%) | <0.001** | 1,870 (19.85%) | 1,806 (18.91%) | 0.27 |
| **Chronic bronchitis, n (%)** | 1,313 (6.87%) | 363 (3.71%) | <0.001** | 343 (3.89%) | 363 (3.75%) | 0.73 |
| **Cancer, n (%)** | 1,961 (10.54%) | 787 (9.38%) | 0.028* | 801 (8.68%) | 785 (9.47%) | 0.17 |
| Note: MVPA: moderate-to-vigorous physical activity. P-values were calculated using survey-weighted statistical tests. Propensity score matching (PSM) was conducted using the 1:1 "nearest" method to balance covariates between groups. * P < 0.05, ** P < 0.001 | | | | | | |

**
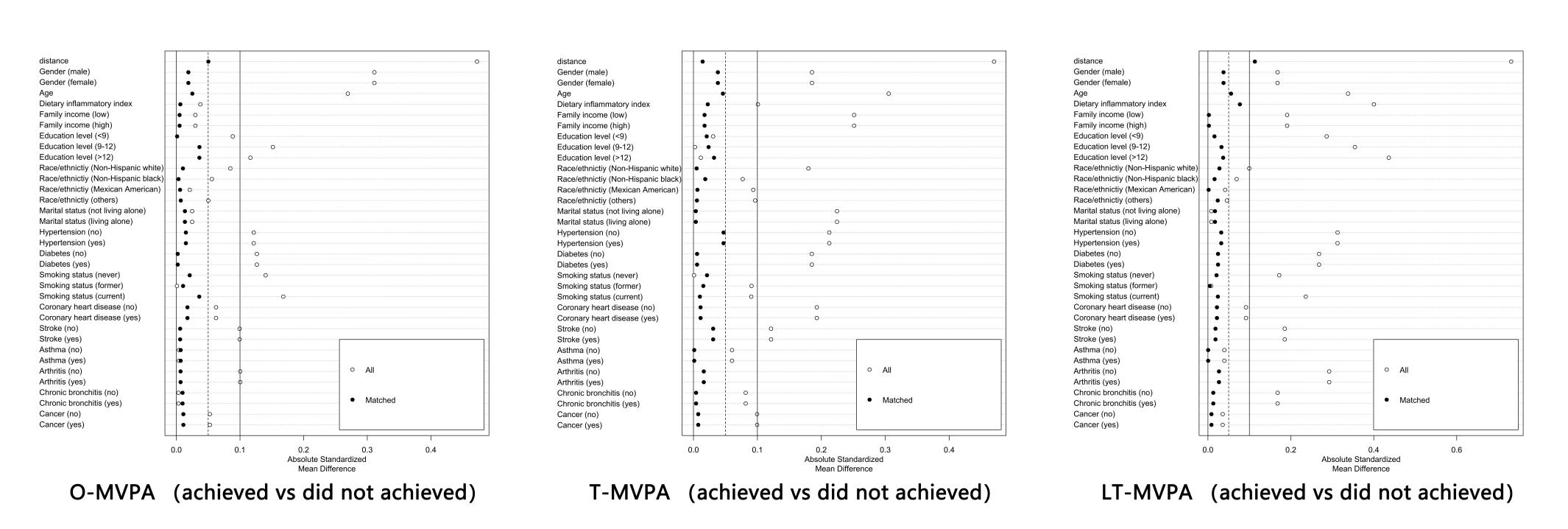
**

**Figure S2.** Propensity score matching (PSM) effect evaluated by love plots. Propensity score matching (PSM) was conducted using the 1:1 "nearest" method to balance all covariates including the Dietary Inflammatory Index (DII) between groups. White dots indicate the standard mean diferences before matching, and black dots indicate that after matching. The dashed line represent an standardized mean differences threshold of 1 for evaluating covariate balance. MVPA: moderate-to-vigorous physical activity, O-MVPA: Occupation-related MVPA, T-MVPA: Transportation-related MVPA, LT-MVPA: Leisure-time MVPA.

| **Table S4. Association between domain-specific MVPA and inflammatory index, after matching** | | | | |
| --- | --- | --- | --- | --- |
|  | **β (95% CI)** | | | |
|  | **Model 1** | **P-value** | **Model 2** | **P-value** |
| **SII** |  |  |  |  |
| **Occupation-related MVPA** |  |  |  |  |
| Did not achieve | Ref |  | Ref |  |
| Achieved | -7.1 (-18 - 4.1) | 0.21 | -7.8 (-19 - 3.6) | 0.18 |
| **Transportation-related MVPA** |  |  |  |  |
| Did not achieve | Ref |  | Ref |  |
| Achieved | -25 (-43 - -7.8) | 0.005* | -24 (-41 - -6.9) | 0.007* |
| **Leisure-time MVPA** |  |  |  |  |
| Did not achieve | Ref |  | Ref |  |
| Achieved | -37 (-49 - -25) | <0.001** | -35 (-47 - -24) | <0.001** |
| **SIRI** |  |  |  |  |
| **Occupation-related MVPA** |  |  |  |  |
| Did not achieve | Ref |  | Ref |  |
| Achieved | -0.01 (-0.05 - 0.02) | 0.35 | -0.02 (-0.05 - 0.01) | 0.25 |
| **Transportation-related MVPA** |  |  |  |  |
| Did not achieve | Ref |  | Ref |  |
| Achieved | -0.08 (-0.14 - -0.02) | 0.007* | -0.08 (-0.14 - -0.02) | 0.009* |
| **Leisure-time MVPA** |  |  |  |  |
| Did not achieve | Ref |  | Ref |  |
| Achieved | -0.09 (-0.14 - -0.05) | <0.001** | -0.09 (-0.13 - -0.05) | <0.001** |
| **NLR** |  |  |  |  |
| **Occupation-related MVPA** |  |  |  |  |
| Did not achieve | Ref |  | Ref |  |
| Achieved | -0.03 (-0.06 - 0.01) | 0.19 | -0.03 (-0.07 - 0.01) | 0.15 |
| **Transportation-related MVPA** |  |  |  |  |
| Did not achieve | Ref |  | Ref |  |
| Achieved | -0.09 (-0.14 - -0.05) | <0.001** | -0.09 (-0.14 - -0.04) | <0.001** |
| **Leisure-time MVPA** |  |  |  |  |
| Did not achieve | Ref |  | Ref |  |
| Achieved | -0.08 (-0.12 - -0.05) | <0.001** | -0.08 (-0.11 - -0.04) | <0.001** |
| **Note:** MVPA: moderate-to-vigorous physical activity. SII: systemic immune inflammation index; SIRI: systemic inflammation response index; NLR: Neutrophil-to-lymphocyte ratio. Propensity score matching (PSM) was conducted using the 1:1 "nearest" method to balance covariates between groups. Model 1 was adjusted for age, sex, race/ethnicity, education level, marital status, and family income. Model 2 was adjusted for age, sex, race/ethnicity, education level, marital status, family income, smoking status, hypertension, diabetes, coronary heart disease, stroke, asthma, arthritis, chronic bronchitis, cancer, and dietary inflammatory index. * P < 0.05, ** P < 0.001 | | | | |
| \| **Table S5. Association between domain-specific MVPA and inflammatory index** \| \| \| \| \| \| --- \| --- \| --- \| --- \| --- \| \|  \| **β (95% CI)** \| \| \| \| \| **Model 1** \| **P-value** \| **Model 2** \| **P-value** \| \| **SII** \|  \|  \|  \|  \| \| **Occupation-related MVPA** \|  \|  \|  \|  \| \| None (0 min/week) \| Ref \|  \| Ref \|  \| \| Any (> 0 min/week) \| -4.4 (-14 - 5.1) \| 0.36 \| -2.9 (-12 - 6.5) \| 0.54 \| \| Achieved (≥ 300 min/week) \| -6.6 (-17 - 4.1) \| 0.22 \| -8.8 (-20 - 1.9) \| 0.11 \| \| **Transportation-related MVPA** \|  \|  \|  \|  \| \| None (0 min/week) \| Ref \|  \| Ref \|  \| \| Any (> 0 min/week) \| -22 (-32 - -12) \| <0.001** \| -18 (-28 - -7.8) \| <0.001** \| \| **Leisure-time MVPA** \|  \|  \|  \|  \| \| None (0 min/week) \| Ref \|  \| Ref \|  \| \| Any (> 0 min/week) \| -48 (-57 - -38) \| <0.001** \| -40 (-50 - -30) \| <0.001** \| \| **NLR** \|  \|  \|  \|  \| \| **Occupation-related MVPA** \|  \|  \|  \|  \| \| None (0 min/week) \| Ref \|  \| Ref \|  \| \| Any (> 0 min/week) \| -0.02 (-0.05 - 0.01) \| 0.22 \| -0.02 (-0.06 - 0.01) \| 0.18 \| \| Achieved (≥ 300 min/week) \| -0.03 (-0.07 - 0.01) \| 0.10 \| -0.03 (-0.07 - 0.00) \| 0.075 \| \| **Transportation-related MVPA** \|  \|  \|  \|  \| \| None (0 min/week) \| Ref \|  \| Ref \|  \| \| Any (> 0 min/week) \| -0.07 (-0.11 - -0.04) \| <0.001** \| -0.06 (-0.09 - -0.02) \| 0.001* \| \| **Leisure-time MVPA** \|  \|  \|  \|  \| \| None (0 min/week) \| Ref \|  \| Ref \|  \| \| Any (> 0 min/week) \| -0.14 (-0.17 - -0.10) \| <0.001** \| -0.12 (-0.15 - -0.08) \| <0.001** \| \| **SIRI** \|  \|  \|  \|  \| \| **Occupation-related MVPA** \|  \|  \|  \|  \| \| None (0 min/week) \| Ref \|  \| Ref \|  \| \| Any (> 0 min/week) \| -0.01 (-0.03 - 0.02) \| 0.54 \| -0.01 (-0.04 - 0.01) \| 0.30 \| \| Achieved (≥ 300 min/week) \| -0.01 (-0.04 - 0.02) \| 0.41 \| -0.02 (-0.04 - 0.01) \| 0.17 \| \| **Transportation-related MVPA** \|  \|  \|  \|  \| \| None (0 min/week) \| Ref \|  \| Ref \|  \| \| Any (> 0 min/week) \| -0.09 (-0.12 - -0.06) \| <0.001** \| -0.07 (-0.10 - -0.04) \| <0.001** \| \| **Leisure-time MVPA** \|  \|  \|  \|  \| \| None (0 min/week) \| Ref \|  \| Ref \|  \| \| Any (> 0 min/week) \| -0.14 (-0.16 - -0.12) \| <0.001** \| -0.11 (-0.14 - -0.09) \| <0.001** \| \| **Note:** MVPA: moderate-to-vigorous physical activity. SII: systemic immune inflammation index; SIRI: systemic inflammation response index; NLR: Neutrophil-to-lymphocyte ratio. Model 1 was adjusted for age, sex, race/ethnicity, education level, marital status, and family income. Model 2 was adjusted for age, sex, race/ethnicity, education level, marital status, family income, smoking status, hypertension, diabetes, coronary heart disease, stroke, asthma, arthritis, chronic bronchitis, and cancer. * P < 0.05, ** P < 0.001 \| \| \| \| \|   **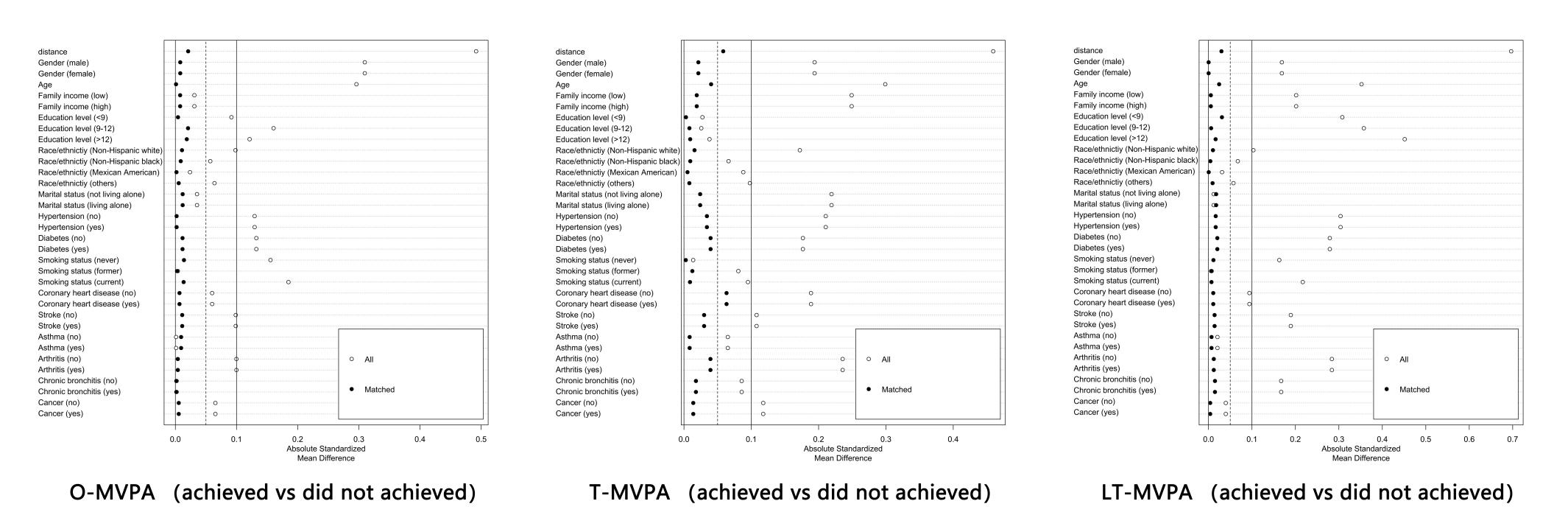**  **Figure 3S.** Propensity score matching (PSM) effect evaluated by love plots. Propensity score matching (PSM) was conducted using the 1:1 "subclass" method to balance covariates between groups. White dots indicate the standard mean diferences before matching, and black dots indicate that after matching. The dashed line represent an standardized mean differences threshold of 1 for evaluating covariate balance. MVPA: moderate-to-vigorous physical activity, O-MVPA: Occupation-related MVPA, T-MVPA: Transportation-related MVPA, LT-MVPA: Leisure-time MVPA.   \| **Table S6. Association between domain-specific MVPA and inflammatory index, after matching** \| \| \| \| \| \| --- \| --- \| --- \| --- \| --- \| \|  \| **β (95% CI)** \| \| \| \| \| **Model 1** \| **P-value** \| **Model 2** \| **P-value** \| \| **SII** \|  \|  \|  \|  \| \| **Occupation-related MVPA** \|  \|  \|  \|  \| \| Did not achieve \| Ref \|  \| Ref \|  \| \| Achieved \| -5.4 (-16 - 5.8) \| 0.31 \| -5.3 (-16 - 5.3) \| 0.32 \| \| **Transportation-related MVPA** \|  \|  \|  \|  \| \| Did not achieve \| Ref \|  \| Ref \|  \| \| Achieved \| -20 (-30 - -9.0) \| <0.001** \| -19 (-31 - -8.2) \| <0.001** \| \| **Leisure-time MVPA** \|  \|  \|  \|  \| \| Did not achieve \| Ref \|  \| Ref \|  \| \| Achieved \| -39 (-48 - -29) \| <0.001** \| -38 (-48 - -28) \| <0.001** \| \| **SIRI** \|  \|  \|  \|  \| \| **Occupation-related MVPA** \|  \|  \|  \|  \| \| Did not achieve \| Ref \|  \| Ref \|  \| \| Achieved \| -0.02 (-0.04 - 0.01) \| 0.26 \| -0.02 (-0.04 - 0.01) \| 0.26 \| \| **Transportation-related MVPA** \|  \|  \|  \|  \| \| Did not achieve \| Ref \|  \| Ref \|  \| \| Achieved \| -0.07(-0.10 - 0.03) \| <0.001** \| -0.06 (-0.10 - 0.03) \| <0.001** \| \| **Leisure-time MVPA** \|  \|  \|  \|  \| \| Did not achieve \| Ref \|  \| Ref \|  \| \| Achieved \| -0.08 (-0.11 - -0.06) \| <0.001** \| -0.08 (-0.11 - -0.05) \| <0.001** \| \| **NLR** \|  \|  \|  \|  \| \| **Occupation-related MVPA** \|  \|  \|  \|  \| \| Did not achieve \| Ref \|  \| Ref \|  \| \| Achieved \| -0.02 (-0.06 - 0.01) \| 0.19 \| -0.02 (-0.06 - 0.01) \| 0.21 \| \| **Transportation-related MVPA** \|  \|  \|  \|  \| \| Did not achieve \| Ref \|  \| Ref \|  \| \| Achieved \| -0.09 (-0.12 - -0.06) \| <0.001** \| -0.08 (-0.11 - -0.05) \| <0.001** \| \| **Leisure-time MVPA** \|  \|  \|  \|  \| \| Did not achieve \| Ref \|  \| Ref \|  \| \| Achieved \| -0.09 (-0.13 - -0.06) \| <0.001** \| -0.09 (-0.13 - -0.05) \| <0.001** \| \| **Note:** MVPA: moderate-to-vigorous physical activity. SII: systemic immune inflammation index; SIRI: systemic inflammation response index; NLR: Neutrophil-to-lymphocyte ratio. Propensity score matching (PSM) was conducted using the "subclass" method to balance covariates between groups. Model 1 was adjusted for age, sex, race/ethnicity, education level, marital status, and family income. Model 2 was adjusted for age, sex, race/ethnicity, education level, marital status, family income, smoking status, hypertension, coronary heart disease, stroke, asthma, arthritis, chronic bronchitis, and cancer. * P < 0.05, **P < 0.001 \| \| \| \| \| | | | | |

|  |
| --- |
